# Effects of a supplemented diet containing 7 probiotic strains (Honeybeeotic) on honeybee physiology and immune response: analysis of hemolymph cytology, phenoloxidase activity, and gut microbiome

**DOI:** 10.1186/s40659-024-00533-x

**Published:** 2024-08-07

**Authors:** Patrizia Robino, Livio Galosi, Alessandro Bellato, Silvia Vincenzetti, Elena Gonella, Ilario Ferrocino, Evelina Serri, Lucia Biagini, Alessandra Roncarati, Patrizia Nebbia, Chiara Menzio, Giacomo Rossi

**Affiliations:** 1 Department of Veterinary Sciences, University of Turin, Turin, Italy; 2 School of Biosciences and Veterinary Medicine, University of Camerino, Matelica, Italy; 3 Department of Agricultural, Forest and Food Sciences, University of Turin, Turin, Italy

**Keywords:** *Apis mellifera ligustica*, Probiotic, Cellular immunity, Intestinal microbiota

## Abstract

**Background:**

In this study, a probiotic mixture (Honeybeeotic) consisting of seven bacterial strains isolated from a unique population of honeybees (*Apis mellifera ligustica*) was used. That honeybee population was located in the Roti Abbey locality of the Marche Region in Italy, an area isolated from human activities, and genetic contamination from other honeybee populations. The aim was to investigate the effects of this probiotic mixture on the innate immunity and intestinal microbiome of healthy common honeybees in two hives of the same apiary. Hive A received a diet of 50% glucose syrup, while hive B received the same syrup supplemented with the probiotics, both administered daily for 1 month. To determine whether the probiotic altered the immune response, phenoloxidase activity and hemolymph cellular subtype count were investigated. Additionally, metagenomic approaches were used to analyze the effects on gut microbiota composition and function, considering the critical role the gut microbiota plays in modulating host physiology.

**Results:**

The results revealed differences in hemocyte populations between the two hives, as hive A exhibited higher counts of oenocytoids and granulocytes. These findings indicated that the dietary supplementation with the probiotic mixture was safe and well-tolerated. Furthermore, phenoloxidase activity significantly decreased in hive B (1.75 ± 0.19 U/mg) compared to hive A (3.62 ± 0.44 U/mg, *p* < 0.005), suggesting an improved state of well-being in the honeybees, as they did not require activation of immune defense mechanisms. Regarding the microbiome composition, the probiotic modulated the gut microbiota in hive B compared to the control, retaining core microbiota components while causing both positive and negative variations. Notably, several genes, particularly KEGG genes involved in amino acid metabolism, carbohydrate metabolism, and branched-chain amino acid (BCAA) transport, were more abundant in the probiotic-fed group, suggesting an effective nutritional supplement for the host.

**Conclusions:**

This study advocated that feeding with this probiotic mixture induces beneficial immunological effects and promoted a balanced gut microbiota with enhanced metabolic activities related to digestion. The use of highly selected probiotics was shown to contribute to the overall well-being of the honeybees, improving their immune response and gut health.

## Introduction

The worldwide importance of *Apis mellifera* as a pollinator has driven several studies aimed at improving the health of their colonies [1–4]. In Italy, the honeybee species *A. mellifera*, specifically the subspecies *A. mellifera ligustica*, play crucial roles in both agriculture and biodiversity. Research has shown that this subspecies has adapted well to Italy’s temperate climate, benefiting from its rich floristic diversity. It is widely distributed across the Italian peninsula and it is also found in regions such as North America, northern and central Europe, and even in isolated locations like Kangaroo Island in Australia and Reunion Island [5, 6].

Understanding the physiological and immunological mechanisms behind these adaptations is essential for effective conservation strategies. In insects, hemolymph is acknowledged as a crucial mediator of nutritional and immunological homeostasis. In addition to transporting nutrients to cells and maintaining body temperature, it also plays a pivotal role in both constitutive and induced immune response [7–9]. The constitutive responses include humoral responses such as prophenoloxidase (proPO) activating system and the cellular responses (e.g., coagulation, phagocytosis, nodule formation, encapsulation), resulting in a rapid elimination of pathogens [10]. Hemocytes have a major role in immune defence in honeybees; specifically, granulocytes are involved in phagocytosis and encapsulation, whereas plasmatocytes act as phagocytes in the presence of antigens [11].

In the honeybee, a key player in immune stimulation is the gut microbiome [9, 12, 13]. The bee microbiome plays a crucial role in the prevention of diseases afflicting their colonies and is crucial in ensuring the proper development of the immune system. Bees have a highly specialized, stable gut microbiota, with a different composition depending on the digestive tract considered (crop, midgut, ileum, rectum), consisting of a small number of abundant species and several low-abundance species [14]. A few bacterial phylotypes constitute the core microbiome, with specific localization for each member in the gut, and a continuous interplay among bacteria and with the host. Since the microbiome is known to prevent pathogen invasion, maintaining its composition unchanged is crucial to keep a correct health status for the bee; conversely, microbiome disruption (dysbiosis) severely affects immunity, metabolism, behavior, and development of the host [13, 15].

An accurate identification of microbes constituting the microbiota is possible through culture-independent approaches [16]. In particular, whole-genome shotgun sequencing (WGS), which uses sequencing with random primers to sequence overlapping regions of a genome, allows us to enumerate and assemble the bacterial species present in the inoculum with high precision, with accurate identification of taxa at the species level, allows us greater identification of bacterial diversity than other methods, and helps us understand the complex interactions between microbes [17, 18].

In recent years, instances of insufficient nutrition for bees, particularly in terms of the protein supply provided by pollen in their natural environment, have become increasingly common [19]. Nutritionally poor diets may derive from reduced floral diversity offered to honeybees in areas with intensive agriculture [20, 21]. Consequently, there has been a rising interest in dietary supplement use, such as probiotic blend, with conflicting opinions. It has been debated how the incorrect selection of probiotic strains may negatively impact the health of honeybees [22].

Although it is a common opinion that administering probiotic bacterial strains improves the health of individual bees and hives, and several commercial probiotics are available for bees, knowledge of probiotic benefits is still to be improved [23]. In recent years, honeybee probiotics have become increasingly purchased by beekeepers because of product claims like being able to *“replenish the microbes lost due to agricultural modifications of honeybees’ environment”* or *“promote optimal gut health”* [24]. However, most bacterial species used in commercial probiotics are not native to bee guts. This can seriously affect the beneficial effect that these bacteria, not deriving from the bee’s gastrointestinal tract, can have on the health of the bee itself. Endogenous bee gut microbes play a role in digestion, detoxification, nutrient conversion, and resistance to pests and pathogens [12]. If the health of bees and gut microbes are functionally codependent, then probiotic supplementation with the use of bacteria that are part of the bee’s intestinal microbiota, which support and restore digestive functions, seems an effective way to improve bee health or at least mitigate the worst effects of disease [25–29]. Regarding the effect of commercially used probiotics on bee health, studies have shown unpromising results. One study found that honeybees fed sugar syrup with *Lacticaseibacillus rhamnosus* (a probiotic) and inulin (a prebiotic) were more susceptible to *Nosema ceranae* infection [30]. Another study indicated that supplementing with the probiotic SuperDFM-HoneyBee™ after oxytetracycline treatment did not restore the honeybee gut microbiota, showing no difference between antibiotic-treated bees with or without the probiotic [24].

This study aimed to assess the effects of a diet supplemented with a complex probiotic preparation derived from a population of wild honeybees. These insects have been living in a remote and isolated area of the Marche Apennines, at an altitude of 950 m above sea level, for about 250 years without any known human interference. In particular, the goal was to evaluate the complex effects of this administration on host physiology, the composition of the intestinal microbiota in qualitative and quantitative terms, intestinal function and modulation of the immune response.

## Materials and methods

### Animal trials

To investigate the effects of the probiotic administration on the innate immune response and microbiome composition in common honeybees (*Apis mellifera ligustica*), two hives of the apiary located at the School of Biosciences and Veterinary Medicine of the University of Camerino (Matelica, Italy), (WGS 84 coordinates, pseudo-Mercator: 43.2589219, 13.0114508) were used. Worker healthy bees were provided with different beekeeper-formulated diets: hive A received a diet comprising 50% glucose syrup while hive B received the same syrup supplemented with Honeybeeotic probiotic mixture. This probiotic blend was formulated using bacterial strains isolated from the gut of a unique population of honeybees living in Roti Abbey area (Matelica, Marche Region, Italy, 950 m a.s.l.) for many years, and it was administered *ad libitum* (1 L/day), for 1 month, between May and June 2022. The probiotic consisted of a mixture of 7 bacteria strains, each adjusted to achieve the same final concentration (2 × 10^11^ CFU/L). Since no consensus existed about the minimal probiotic concentration, we chose this in line with previous studies that evidenced probiotic effects with both lower [31, 32] and higher [33] concentrations. The isolates included in the mixture were *Apilactobacillus apinorum* (DSM 34547), *Lactiplantibacillus plantarum* (DSM 33923), *Lactiplantibacillus fabifermentas* (DSM 34546), *Lactiplantibacillus plantarum* (DSM 34454), *Lactiplantibacillus plantarum* (DSM 34542), *Lactiplantibacillus plantarum* (DSM 34500), and *Lactiplantibacillus plantarum* (DSM 34499). All strains were deposited at the DSMZ biobank (www.dsmz.de.), where they are stored for future use.

### Hemolymph cytology

At the end of the trial (T1), a sample of 25 μL of haemolymph was collected from ten foraging honeybees per hive. Briefly, honeybees were anaesthetised by CO2 inhalation, and a glass capillary was introduced in the *sinus dorsalis*, between the third and fourth abdominal segments. Each sample was smeared on a slide for cytological examination, air dried and stained with May Grünwald Giemsa for smears – kit (Histo-Line Laboratories, Milano, Italy). Stained preparations were investigated with an optical microscope (Leica DM2500, Wetzlar, Germany) using 20x, 40x and 100x magnifications. Hemocytes were quantified by counting them on each slide and classified based on their morphology as hemocytes, plasmatocytes, granulocytes and oenocytoids [11].

### Phenoloxidase activity determination on haemolymph extracts

Using the same approach, 25 μL samples of haemolymph were collected from ten honeybees per hive at T1. Samples were pooled and stored without any buffer at -80 °C until use. Subsequently, they were thawed on ice, vortexed briefly, and centrifuged with a microfuge to remove cell debris (15,000 rpm, 20 min). The obtained supernatant was diluted 1:10, 1:20, and 1:40, in 10 mM phosphate buffer saline (PBS), pH 7.2. PO activity has been carried out according to published protocols [10, 34], with some modifications; all determinations have been performed in triplicate. The enzymatic activity was detected in the haemolymph samples by a continuous spectrophotometric assay measuring the absorbance at λ = 475 nm of the color reaction product (dopa-chrome) formed by the oxidation of the substrate (L-Dopa. o-diphenol, Sigma Aldrich, USA). L-Dopa stock solution was prepared by dissolving 3 mg of the substrate in 1 ml of ultrapure water). Each sample test was performed in 250 μL quartz cuvettes, the reaction mixture contained 120 μL Phosphate Buffered Solution (PBS) pH 7.2, 60 μL L-Dopa (final concentration 4.5 mM), and 20 μL of each diluted pool, the total volume was 200 μL. The reaction was allowed to proceed at 30 °C in a spectrophotometer for 6 min. In parallel, a control test was carried out by preparing the reaction mixtures described above plus 10 mM diethyl thiocarbamate (DETC, Sigma Aldrich, USA) which is a specific PO inhibitor [35]. In addition, a substrate auto-oxidation control was performed, replacing the hemolymph sample with 20 μL of ultrapure water. The values obtained for these controls were subtracted from the test. One unit of enzyme activity is defined as the amount of enzyme which catalyzes the conversion of 1 μm of L-Dopa in dopachrome. The molar extinction coefficient for dopachrome at λ = 475 nm is 3600 M^− 1^ cm^− 1^. The total protein content on hemolymph samples has been determined by the Bradford method [36].

### DNA extraction and metagenomic sequencing

The honeybees required for the metagenomic analyses were taken from the two hives before the start of the experiment (T0) and at the end of the experiment (T1), and immediately frozen at -80 °C. The insects were dissected to obtain separate samples from four tracts of the digestive system, namely, crop, midgut, ileum, and rectum according to Callegari et al. [37]. Gut dissection was performed in PBS under a stereomicroscope in sterile conditions using sterile forceps and needles. Whole guts were frozen at -20 °C for ten minutes before separating the compartments, which was done using a scalpel sterilized between every cut. Specimens were discarded whenever any portion of the gut was released into the intestinal liquid during the dissection of the fresh tissues or separation of the frozen tissues.

Pools of 15 honeybees/hive were prepared for each gut compartment to have more material from which to extract DNA. Microbial DNA extraction was performed using a commercial kit (QIAamp PowerFecal Pro DNA, Qiagen, Hilden, Germany), on 16 pools of the different digestive system tracts according to the manufacturer’s instructions. Eluted nucleic acids were quantified by NanoDrop instrument (Celbio, Milan, Italy) and DNA samples were standardized at ≥ 10 ng/μL, ≥ 200 ng, and stored frozen (− 20 °C) until use. Whole metagenomics (150 bp paired-end reads) was performed on a NovaSeq Illumina platform by the Novogene Biotechnology Company Ltd (Cambridge, UK).

### Bioinformatics and statistical analysis

Data related to haemolymph cytology were analysed using GraphPad Prism 9 software (GraphPad Software, Inc., La Jolla, CA, USA). All data are presented as the means ± standard deviation (SD) and were first checked for normality using the Shapiro-Wilk normality test. Differences in hemocytes, plasmatocytes, granulocytes and oenocytoids count from the hemolymph of bees belonging to the control and the treated group were analyzed using a Two-Way Analysis of Variance (ANOVA) followed by Sidak’s multiple comparison test. A *P* < 0.05 was considered significant.

The whole metagenome data analysis was performed as follows. The obtained reads were mapped to the draft genome of *Apis mellifera* by Bowtie2 [38] in end-to-end sensitive mode. AdapterRemoval [39] was then used to remove low-quality reads with ambiguous bases and sequences less than 50 bp. Clean reads were then used for functional analysis with HUMAnN3 [40] using default parameters. Next, the gene abundance matrix was further collapsed by the Kyoto Encyclopedia of Genes and Genomes (KEGG) Orthology term via the “humann_regroup_table” function provided within HUMAnN3. For the same set of metagenomes, we used MetaPhlAn3 [41] to estimate the relative abundance of taxa at the species level.

The relative abundance of SGBs (Species-level genome bins) was analyzed to identify differences using the Kruskal-Wallis test. Alpha- and beta-diversity were calculated. Alpha diversity was evaluated through the calculation of Shannon’s index on SGBs’ relative abundance and compared by Wilcoxon rank sum and Kruskal-Wallis tests. Beta diversity was evaluated using three different indexes, Sorensen’s (B_sor_), Jaccard’s, and Harte e Kinzig’s index. B_sor_ was then used for comparison through Kruskal Wallis and Wilcoxon rank-sum tests.

## Results and discussion

### Haemolymph cytology

**Fig. 1:**
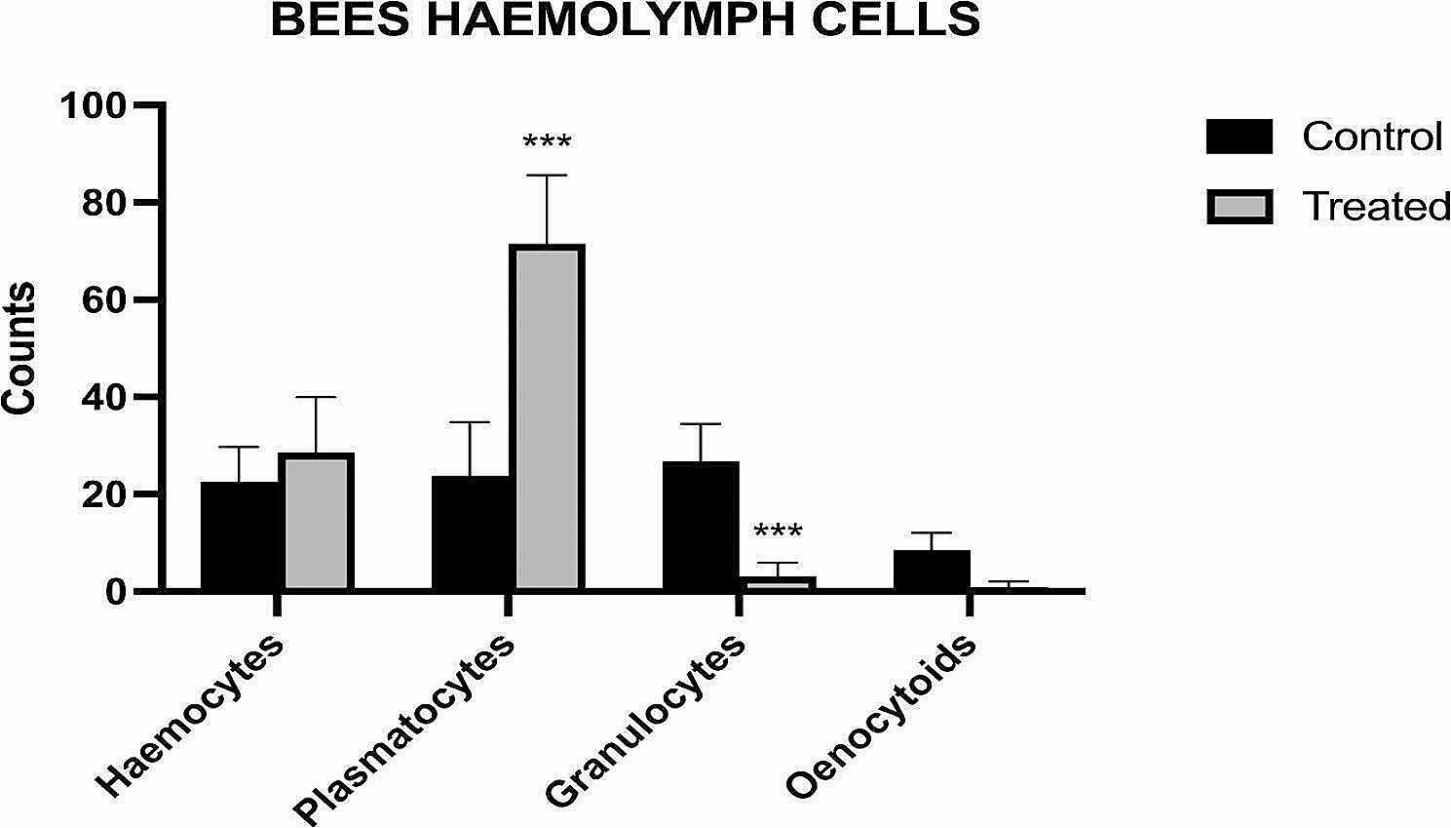
Cytological smears from haemolymph samples showed a significant difference in plasmatocyte and granulocyte cell counts between the two populations of bees (hive A, control, vs. hive B, treated). Bees belonging to hive A showed a higher granulocytes cell count (*P* < 0.01) compared to bees treated with probiotics that showed a significant amount of plasmatocytes (*P* < 0.01). ***P < 0.01

**Fig. 2:**
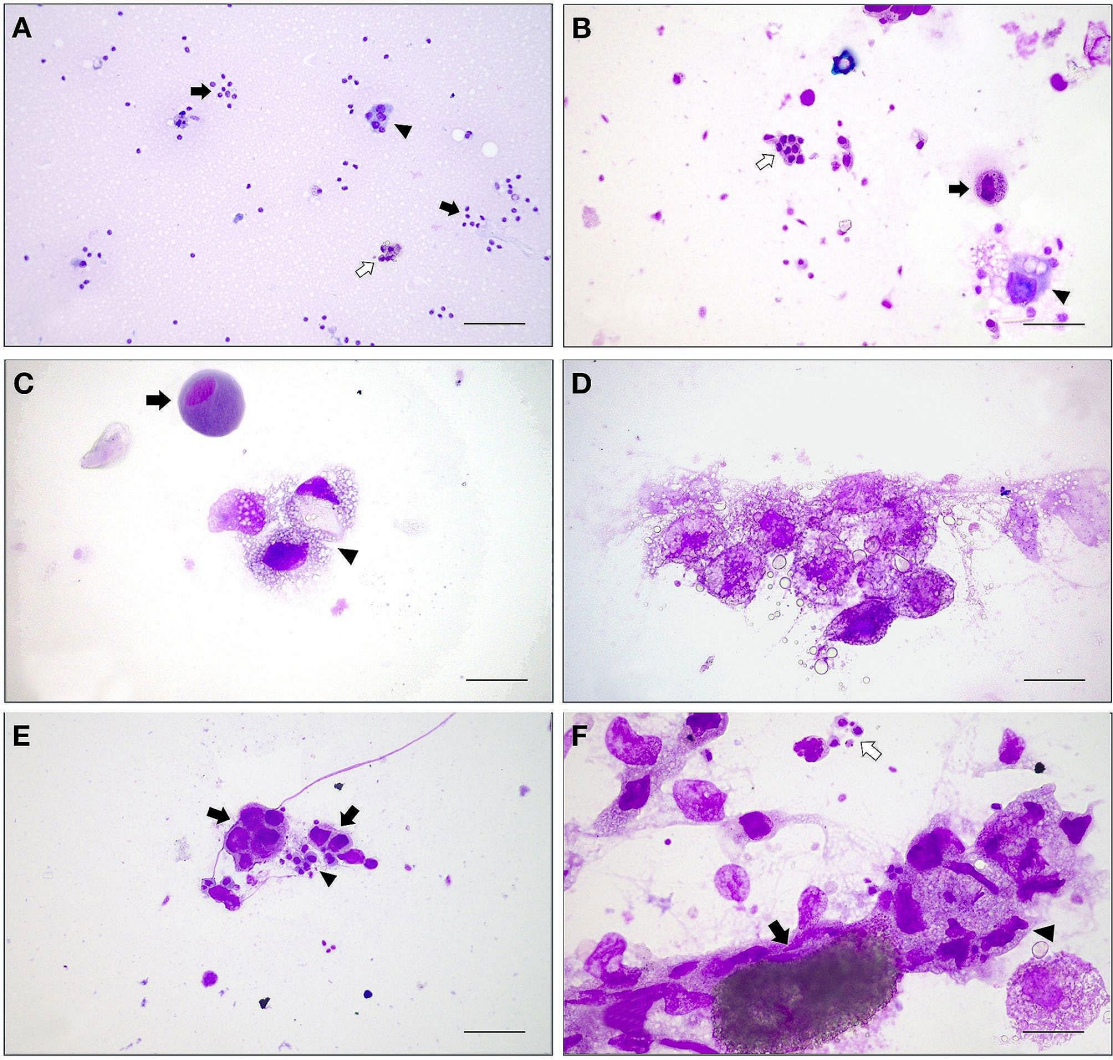
Air dried and May Grünwald Giemsa-stained cytological slides from honeybee haemolymph. **A**) some hemocytes (arrows), a group of plasmatocytes (arrowhead), and a group of granulocytes (open arrow) are present; scale bar = 200 μm. **B**) some hemocytes (open arrow), a granulocyte with some dark colored granules of PO (arrow), and a plasmatocyte phagocytizing a foreign particle (arrowhead); scale bar = 100 μm. **C**) a group of “activated” plasmatocytes (arrowhead), and a single, inactive plasmatocyte (arrow); scale bar = 50 μm. **D**) a group of activate and “phoamy” plasmatocytes during phagocytosis; scale bar = 50 μm. **E**) some small hemocytes (arrowhead), and a group of plasmatocytes (arrows) at different stages of activation; scale bar = 100 μm. **F**) Small hemocytes (open arrow), a big and pigmented oenocytoid (arrow) with a group of phagocytizing plasmatocytes (arrowhead); scale bar = 50 μm

In agreement with previous literature [42, 43], also in this study plasmatocytes and hemocytes represented the most numerous cell sub-types of all in the hemolymph of both groups of bees, showing values around 70% for plasmatocytes and 30% for prohemocytes. Cytological smears from hemolymph samples showed a significant difference in plasmatocyte and granulocyte cell counts between the two populations of bees (hive A vs. hive B) (Figs. 1 and 2). In particular, the bees belonging to hive A showed a higher granulocyte cell count (*P* < 0.01), to bees treated with probiotics which showed a significant amount of plasmatocytes (*P* < 0.01). The hemocyte populations belonging to the two bees’ populations were not statistically different if compared using a Two-Way Analysis of Variance (ANOVA) followed by Sidak’s multiple comparison test (*P* > 0.05). Table 1 reports the mean percentages among the replicates of each cell type identified within the hemocyte population of each experimental group. The highest concentration of granulocytes was observed in bees from hive A (mean = 17 cells, min-max: 5–33), while plasmatocytes were higher in bees from hive B (mean = 78 cells, min-max: 49–85). The oenocytoides were observed only in the untreated bees from hive A (mean = 8 cells, min-max: 4–15).

**Table 1:** Hemocyte evaluation in honeybees: comparing hive A (control) and hive B (probiotics) at T1

Cell types	Hive A	Hive B
Hemocytes	30 (11–36)	35 (22–44)
Plasmatocytes	49 (4–56)	78 (49–85)
Granulocytes	17 (5–33)	5 (2–9)
Oenocytoids	8 (4–15)	0 (0–1)

Results are expressed as means values (range: minimum-maximum) from 10 insects per hive

Regarding the analysis of the haemocyte pattern, these results show that bees from hive B, that were given probiotic dietary supplementation, differ significantly compared to the untreated control hive (hive A). Specifically, the concentration of plasmatocytes was notably higher in hive B, while granulocyte levels were significantly elevated in the hemolymph of hive A.

Since PO activity is mainly carried out by granulocytes and oenocytoids, these data are in line with the PO activity of hemolymph (see next paragraph). However, the PO activity and the number of “active” granulocytes appeared very low in the hemolymph of hive B bees, while the presence of plasmatocytes was significantly higher. This could indicate that bacterial stimulation, such as that induced by the probiotics composing the Honeybeeotic mixture, can effectively stimulate the cell-mediated response and phagocytosis, rather than the humoral response. It is known that plasmatocytes act as phagocytes only in the presence of the antigens [11]; their action is very specific, and their intervention is secondary to the induced stimulus by other haemocytes. When pathogens are detected by hemocytes, these surround them and release chemoattractant proteins that attract plasmatocytes, forming a “plasmatocyte wall” capable of neutralizing and destroying the pathogen itself, through active phagocytosis [44]. These data are very interesting as they appear in line with observations in other animal species and in humans, where probiotic treatment increases phagocytosis and macrophage-specific cell-mediated action [45]. Haemocytes are involved in coagulation and defense activities such as the synthesis of phenoloxidase, nitric oxide and the aggregation protein (hemokinin) [46–48].

In our study, however, the increase in plasmatocytes in honeybees treated with a probiotic mixture indicates a direct stimulation of this treatment on the cell-mediated response. It could be hypothesized instead that the presence of granulocytes and oenocytoids in untreated bees is due to the synthesis of bioactive compounds, such as PO, which are more expressed in stressed bees with greater stimulation of the innate response [46]. Oenocytoids, which were not found in bees treated with probiotics blend, contain cytoplasmic precursors of phenoloxidase; in fact, bees in hive B had the lowest phenoloxidase activity, confirming the role of these cells in PO production [49].

### Phenoloxidase activity determination on haemolymph extracts

**Fig. 3:**
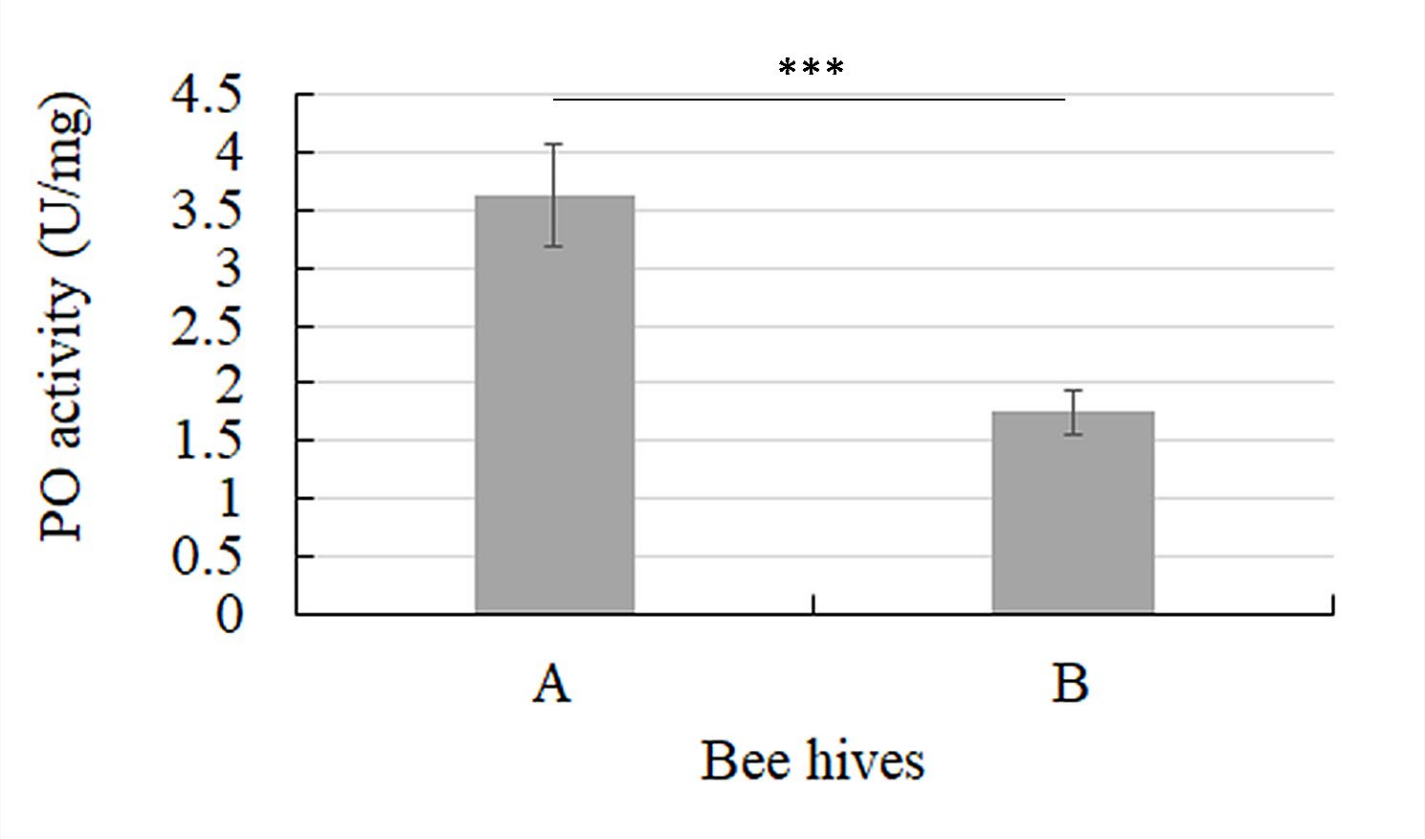
Phenol oxidase (PO) specific activity (U/mg) calculated on the haemolymph samples collected from 20 honeybees of two different hives (A, B) treated as described in the Material and Methods section. *** *P* < 0.005

The PO specific activity in the haemolymph samples coming from the two different bee hives was determined. Results, shown in Fig. 3, indicated that hive B, which received the probiotic Honeybeeotic, showed a significantly reduced PO activity compared to hive A, which received 50% glucose syrup alone (*P* < 0.005). In a recent study it was observed that in honeybees, PO activity is modulated because of stress factors such as pathogens [11]. These researchers evaluated PO activity in the honeybee hemolymph during three stress degrees: naturally infected bees (with deformed wings virus, DWV), PBS-injected bees, and artificially DWV superinfected bees. As a result, they found the lowest PO level in the PBS-injected group and the highest level in the DWV honeybees since the continuous stress condition induced by the superinfection affects the progressive PO synthesis mechanism activation.

In light of these considerations, it could be hypothesized that the probiotics induce a beneficial state that makes the activation of PO unnecessary and therefore it is maintained at low levels, resulting in a significantly lower activity of PO in the hemolymph of honeybees treated with the probiotic Honeybeeotic (hive B). As observed in other animal species [50, 51], in our study the results suggest that probiotics play an important role in the regulation of the immune response, limiting the activation of typical and non-specific factors of innate immunity, also activated in stressful conditions such as PO enzymatic activity. Importantly, the probiotics increase the activation of a greater number of specialized cells for a much more active and effective phagocytic response against specific *noxae* [29].

Finally, our results confirmed a dependent relationship between the probiotics and the type of cellular immune response rather than innate and linked to phenoloxidase activity. Regarding the effect of probiotic supplementation on honeybee’s microbiome composition, according to several studies characterizing the microbiota in bees and its compartmentalization in various digestive system tracts [52–54], metagenomic analysis revealed a characteristic composition of the bee microbiota (core) both before and after probiotic treatment.

### Microbiome profile of the honeybees

**Fig. 4:**
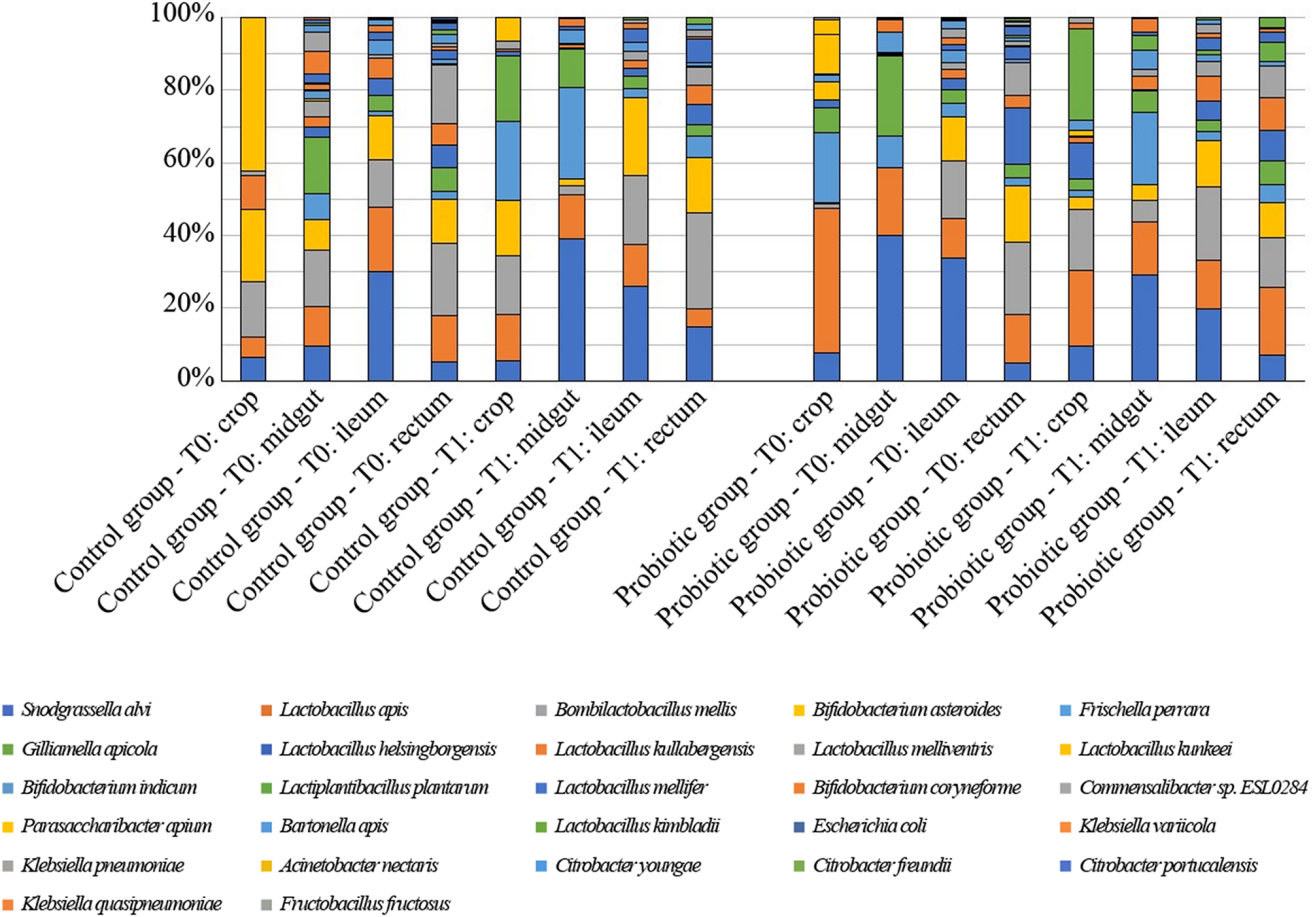
Taxonomy analysis of honeybees. The plot shows the distribution of taxa along time, tract and treatment. Only taxa with an incidence above 0.5% in at least 1 sample are shown

Approximately 60 bacterial species were identified by shotgun NGS sequencing in the honeybee gut compartments, as shown in Fig. 4. In bees receiving probiotic supplementation, the species with > 5% prevalence were: in the crop, *Lactiplantibacillus plantarum* (25.2%), *Lactobacillus apis* (20.7%), *Bombilactobacillus mellis* (17.0%), *Lactobacillus helsingborgensis* (9.8%), *Snodgrassella alvi* (9.6%); in the midgut, *S. alvi* (29.2%), *Frischella perrara* (19.9%), *L. apis* (14.6%), *Giliamella apicola* (6.1%), *B. mellis* (5.7%), *Bifidobacterium indicum* (5.1%); in the ileum, *B. mellis* (20.2%), *S. alvi* (19.7%), *L. apis* (13.5%), *Bifidobacterium asteroides* (12.8%), *Lactobacillus kullabergensis* (6.7%), *L. helsingborgensis* (5.3%); and in the rectum, *L. apis* (18.5%), *B. mellis* (13.6%), *B. asteroides* (9.7%), *L. kullabergensis* (8.9%), *L. helsingborgensis* (8.7%), *Lactobacillus melliventris* (8.6%), *S. alvi* (7.2%), *G. apicola* (6.3%), *L. plantarum* (5.3%).

As shown in Table 2, core microbiota components were conserved in both hives from T0 to T1, with some positive or negative variations. In particular, except for *G. apicola*, all other components’ proportions increased in the crop. Both Firm-4 and Firm-5 Lactobacilli increased in the midgut and ileum and decreased in the rectum. *B. asteroides* increased in the midgut, too. In the rectum, *S. alvi* and *G. apicola* increased.

**Table 2:** Temporal changes in core microbiota between T0 and T1 across bee digestive samples in hives a and B

Taxa		sample							
		Crop		Midgut		Ileum		Rectum	
		Hive A	Hive B	Hive A	Hive B	Hive A	Hive B	Hive A	Hive B
	*Snodgrassella alvi*	↓	↑	↑	↓	↓	↓	↑	↑
	*Gilliamella apicola*	↓	↓	↓	↓	↓	=	↓	↑
	*Bifidobacterium asteroides*	↓	↑	↓	↑	↑	=	↑	↓
Lactobacillus Firm-4	*B. mellis*	=	↑	↓	↑	↑	↑	↑	↓
	*L. mellifer*	=	↑	↑	↑	↑	↑	↑	=
Lactobacillus Firm-5	*L. helsingborgensis*	↑	↑	↓	=	↓	↑	=	↓
	*L. melliventris*	↑	↑	↓	↑	↑	↑	↓	=
	*L. kimbladii*	=	↑	↓	↑	=	=	↑	↑

↑ indicates a quantitative increase in bacterial abundance from T0 to T1; ↓ indicates a quantitative decrease in bacterial abundance from T0 to T1; = indicates no change in bacterial abundance from T0 to T1

This result confirms that probiotic supplementation did not induce adverse variations for the host, not compromising the delicate balance of the intestinal environment, which could lead to detrimental effects. Overall, the data obtained confirm an increase in the relative abundance of *Lactobacillus Firm-4* and *Firm-5*, as well as *Lactiplantibacillus plantarum*, *Lactobacillus apis*, and *Lactiplantibacillus paraplantarum*, of probiotic origin.

It must be pointed out that the absence of statistically significant results in some of the analyses performed may be partly due to the low sample size. However, this does not diminish/devalue the importance of the description made of the “core” microbiota and the considerations drawn in this work, as they are supported by an abundant scientific bibliography [12, 37, 52–54], as well as the data relating to metabolic function and evidence of phenoloxidase activity.

#### Alpha diversity

Alpha diversity was evaluated through the calculation of Shannon’s index on taxa relative abundance, whose mean ranged from 2.11 to 2.44 in the digestive system tracts of bees that received probiotic supplementation (Table 3).

**Table 3:** Alpha diversity of microbiota between T0 and T1 across honeybee digestive samples in hives a and B

	T0		T1	
HIVE A (Control)	**Digestive tract**	**Shannon’s index**	**Digestive tract**	**Shannon’s index**
	Crop	1.59	Crop	1.99
	Midgut	2.66	Midgut	1.73
	Ileum	2.13	Ileum	2.07
	Rectum	2.47	Rectum	2.31
HIVE B (Probiotic)	**Digestive tract**	**Shannon’s index**	**Digestive tract**	**Shannon’s index**
	Crop	1.86	Crop	**2.11**
	Midgut	1.60	Midgut	**2.12**
	Ileum	2.17	Ileum	**2.32**
	Rectum	2.46	Rectum	**2.44**

The table reports the mean Shannon’s index for alpha diversity calculated on the taxa included in the microbiota of honeybees at the beginning of the trial (T0) and after receiving or not the probiotics (T1)

This result suggests an enrichment of the microbiota after probiotic administration. Overall, we observed an increase in alpha diversity in the treatment groups compared with the control group. Alpha diversity increased from T0 to T1 in all digestive system tracts of hive B (treated), while it increased only in the crop in hive A (control), decreasing or remaining steady (rectum) in the other digestive system tracts. The increment was partly due to the bacterial species administered with probiotics, meaning that the administered species were able to colonize the bee’s digestive system. *Lactiplantibacillus plantarum* and *Lactobacillus apis* relative abundance, which was not different between hive A and B at T0, differed at T1. On the contrary, there was no difference between the control and treatment group at T1 for *Lactobacillus kullabergensis* and *Parasaccharibacter apium*, but there was at T0.

**Fig. 5:**
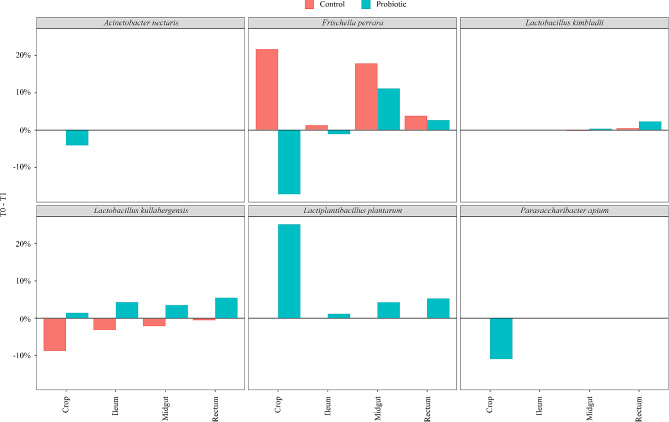
Significant taxa prevalence changes from T0 to T1 per anatomical tract of the digestive tract in hive A (control, red) and hive B (control, blue)

Comparing the change of microbes from T0 to T1, it was possible to highlight those varying the most. Figure 5 shows the taxa proportions that changed significantly from T0 to T1 in every anatomical tract of the digestive system. *Acinetobacter nectaris*, *Frischella perrara*, and *Parasaccharibacter apium* significantly decreased in the crop of bees receiving probiotics, while increasing (*F. perrara*) or remaining stable in the control group. *P. apium* is a potential opportunistic pathogen that can jeopardize the functionality of the intestinal biofilm, with deleterious effects for the host [55]. Also, the proportion of *F. perrara* increased in the midgut and rectum in both the control group and, albeit less so, in the treatment group. The smaller relative increase observed in the treated group could depend on the greater variety of taxa. *F. perrara* is a microorganism strictly involved in bees’ immune response, and its decrement is in line with reduced activation of the immune system thanks to the enhanced fitness of the host, likely induced by the probiotic [56].

Results suggest that some lactobacilli species increased significantly after probiotic administration. In particular, *L. kimbladii*, *L. kullabergensis*, and *L. plantarum* proportions increased significantly in rectum (*L. kimbladii*) or all portions of the gut of bees treated with probiotics.

**Fig. 6:**
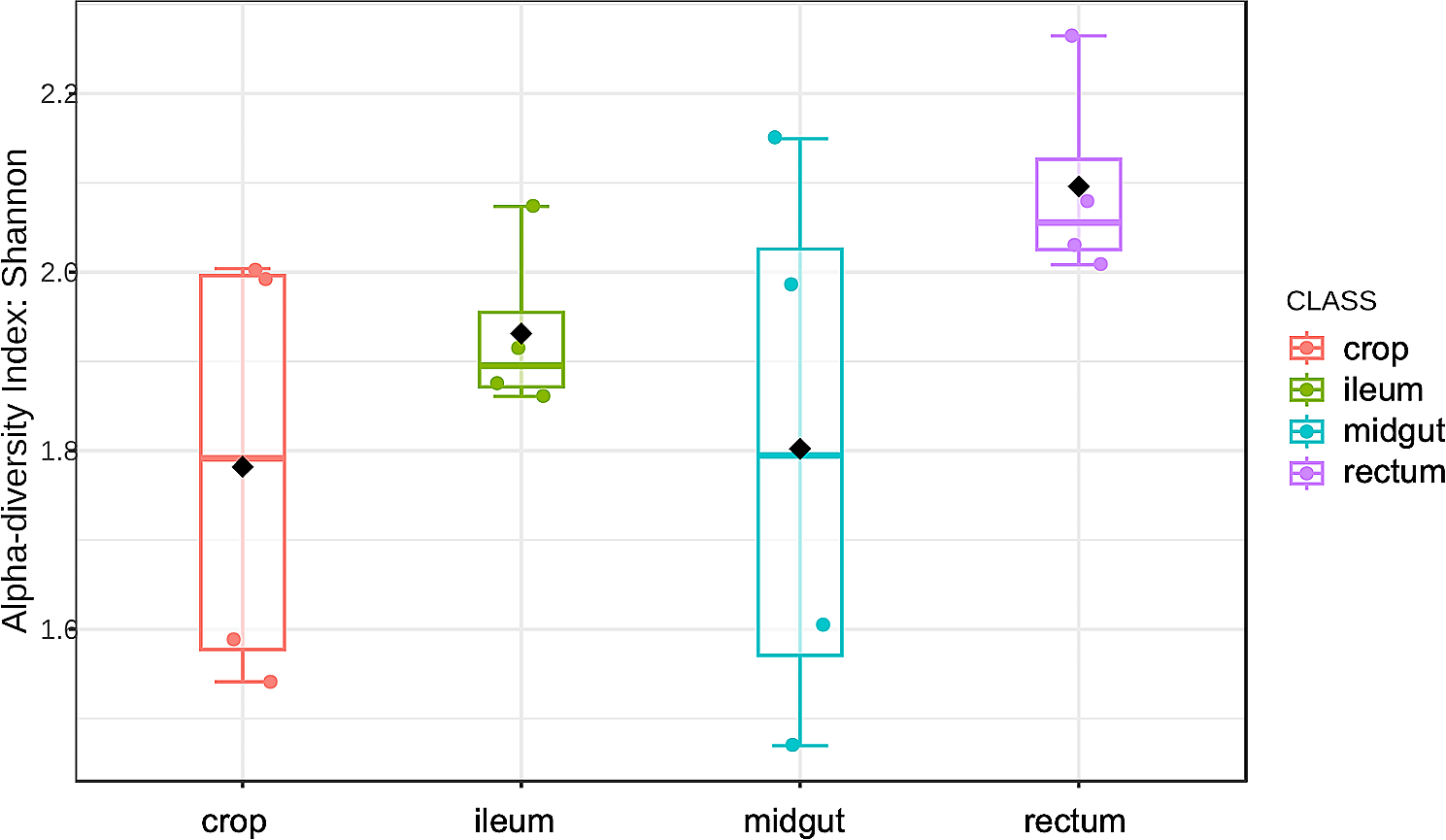
Box plot of the alpha-diversity of each anatomical tract

In addition, comparing Shannon indexes of the different anatomical tracts, albeit not significant (*p* = 0.134), it was observed that ileum and rectum had a greater heterogeneity of distribution of bacterial taxa, accordingly with the expectation that ileum and rectum are the tracts with the greatest abundance of microorganisms [14] (Fig. 6).

#### Beta diversity

Crop microbiota did not differ significantly between control and treatment group (B_sor_ = 0.2), but several taxa were found only after probiotic administration, namely *Bifidobacterium coryneforme*,* Bifidobacterium indicum*,* Commensalibacter spp ESL0284*,* Lactobacillus kimbladii*,* Lactobacillus mellifer*, and *Lactiplantibacillus plantarum.* On the contrary, no SGBs was observed only in control group. In midgut (B_sor_ = 0.1), *L. plantarum* was found only in treated bees, while *Commensalibacter sp ESL0284* and *Morganella morganii* were exclusively found in the control group. Similarly, in ileum (B_sor_ = 0.1), *L. kunkeei* and *L. plantarum* were found only in treated bees, while *Enterobacter mori*,* Leuconostoc mesenteroides*,* Morganella morganii*, and *Providencia rettgeri* were exclusive findings of control group. The highest heterogeneity was observed between rectum samples (B_sor_ = 0.3), where *Citrobacter pasteurii*,* Citrobacter youngae*,* Enterobacter mori*,* Escherichia coli*,* Kosakonia cowanii*,* Lactiplantibacillus paraplantarum*,* Lactiplantibacillus plantarum*,* Leclercia adecarboxylata*,* Pantoea agglomerans*,* Serratia marcescens*,* Serratia ureilytica*, and *Tatumella ptyseos* were the taxa found only in treatment group, while *Citrobacter freundii*,* Citrobacter portucalensis*,* Fructobacillus fructosus*,* Klebsiella aerogenes*, and *Parasaccharibacter apium* were observed in control group only.

To sum up, no significant differences were observed in terms of microbiota between hive A and hive B or as a function of the digestive system tract, either in terms of alpha or beta biodiversity. This is in agreement with previous observations, which indicated similar diversity levels for different gut compartments even if the bacterial composition changes [37], independent of the original family. The absence of significant differences suggests that the feeding integration does not interfere with the core microbiome, avoiding the risk of probiotic-induced dysbiosis. It is possible to speculate on the variations in microbiota as a function of time of sampling, digestive system tract and treatment. The absence of significant differences suggests that the microbiota remains fairly stable in terms of relative abundance, so dietary supplementation with probiotics does not appear to present a risk of potentially harmful dysbiosis conditions developing.

The set of results supports the hypothesis of a beneficial effect on the health of honeybees. The relative increase in abundance of lactobacilli suggests an immune contribution, linked to receptor competition and production of antibacterial substances, conferring greater resistance to the host [12, 57]. Noteworthy is the relative decrease of *F. perrara*, a component of the honeybee gut microbiota, which has a very intense stimulating function on the melanization process [56]. Like all mechanisms involved in the immune system, if the response were excessive, it would be harmful to the host itself and no longer protective. Specifically, the bacteria responsible for fermenting carbohydrates present in the diet, genera *Lactobacillus* and *Bifidobacterium*, are both favored by probiotic intake. Furthermore, these genera possess phosphotransferases implicated in membrane transport function, essential for sugar absorption and energy storage processes [12–14].

### Metagenomic function

**Fig. 7:**
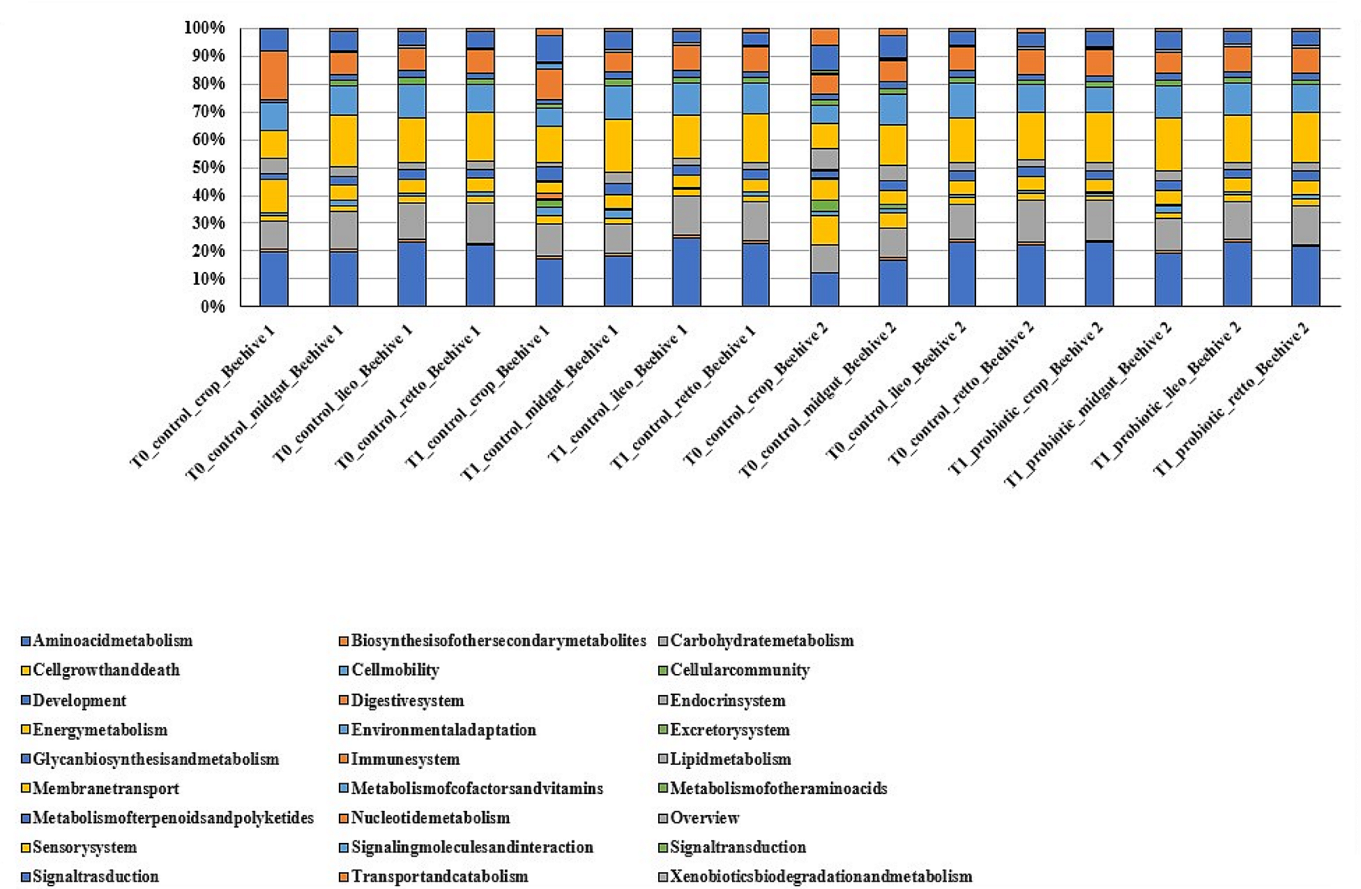
Functional classification of honeybee microbiome. Functional classes were determined according to the first level of the KEGG annotations

The Kyoto Encyclopedia of Genes and Genomes (KEGG) analysis assigned 2192 genes to 27 pathways, and the results gave a highly integrated picture of the global bee microbiome, divided into the four digestive system tracts (Fig. 7).

Considering level 2 of the annotations, the most abundant KEGG categories were metabolism of carbohydrates, amino acids, energy, membrane transport, cofactors and vitamins, nucleotides, polyketides and terpenoids and signal transduction.

Considering the individual digestive system tracts, we can observe that in the crop and midgut the pathways that increased most in hive B, between T0 and T1, are those related to amino acid metabolism, carbohydrate metabolism, nucleotide metabolism and membrane transport; in the crop also cofactor and vitamin metabolism. In the ileum, the same pathways named above are decreased. In the rectum, the metabolic pathways remain relatively stable.

The metabolic functions that increased in some gut compartments in connection with probiotic ingestion are related to nutritional metabolism and interaction with the host, a mechanism that is carried out via membrane transport. It can be assumed, therefore, that the metabolites produced are also transferred to the host and not merely used by the gut microbiota for its functions. This suggests that there is an acquisition of the probiotic by the honeybee and not just a transient effect that will wear off. Considering that the crop is where the microorganisms carried by the probiotic are supposed to settle [58], the results are consistent with what is expected. On the other hand, the increase in metabolic pathways predominantly in the crop and midgut may also be attributable to the increased abundance of certain components of the core microbiota observed in these digestive system tracts. It can therefore be concluded that the gut microbiota acts in synergy with the administered probiotic, enhancing the beneficial effect.

As for metabolic activity, the results obtained are consistent with expectations, showing an increase in metabolic pathways in the crop, where the lactobacilli carry out most of their functions, in accordance with the literature [25, 59]. Considering that one of the metabolisms that increases in this compartment is membrane transport, it can be assumed that the probiotic is not only used for the benefit of the gut microbiota, but there is also an effective passage to the host.

## Conclusions

Our study was born from the increasingly relevant need to safeguard the health of bees, considering the important role they play as bioindicators of the health of the ecosystem and as pollinators. Overall, modulation of the gut microbiome through the use of specific probiotics can be considered a successful strategy to improve the efficiency and well-being of this insect, allowing for more resilient and healthy colonies. As demonstrated in other animal species, the use of specific probiotic bacterial strains can be a useful strategy to increase the immune response of bees too. In conclusion, these results can demonstrate a higher nutritional efficiency of the honeybee and significant modulation of the immune system and digestive microbiota, leading to an overall balanced status capable of providing benefits to the host. Future research will focus on evaluating the efficacy of this probiotic mixture on a production scale, assessing the effects on honey after the use of these native probiotics on honey bees. For instance, a comprehensive characterization of additional immune-related processes could reveal possible protective effects towards specific pathogens or other stressors. Another important focus will be metabolomics studies, concentrating on the transformation metabolite processes in bees fed with native probiotics.

## Data Availability

All data are included in the manuscript.

## Abbreviations

μL: Microliter
bp: Base pairs
CFU/g: Colony forming units per gram
KEGG: Kyoto Encyclopedia of Genes and Genomes
mg: Milligram
min: Minute
ml: Milliliter
ng: Nanogram
nm: Nanometer
U: Unit
